# Design and rationale of the ATtune Knee Outcome Study (ATKOS): multicenter prospective evaluation of a novel uncemented rotating platform knee system

**DOI:** 10.1186/s12891-021-04493-1

**Published:** 2021-07-15

**Authors:** Rachid Rassir, Inger N. Sierevelt, Marjolein Schager, Peter A. Nolte, the ATKOS group, Maarten V. Rademakers, Diederik A. Vergroesen, Paul Spruijt, Niels R. A. Baas, Remko J. A. Sonnega, Paulien M. van Kampen, Herman Lacroix, Wiebe C. Verra, Christiaan P. van Lingen, Tim A. E. J. Boymans, Peter Z. Feczkó, Liesbeth Jütten-Brouwer, Joris A. Jansen, Hans Erik Henkus, Menno R. Benard, Geert Meermans

**Affiliations:** 1 Spaarne Gasthuis, Spaarnepoort 1, 2134 TM Hoofddorp, The Netherlands; 2 Xpert Orthopedie Amsterdam/SCORE (Specialized Center of Orthopedic Research and Education), Laarderhoogtweg 12, 1101 EA Amsterdam, The Netherlands

**Keywords:** Total knee arthroplasty, Attune, Uncemented, Survivorship, Patient reported outcome measures, Sport, Work, Pain catastrophizing, Pain self-efficacy

## Abstract

**Background:**

Total Knee Arthroplasty (TKA) remains the gold standard for treatment of debilitating symptoms of knee osteoarthritis (OA). Even though providing satisfactory results for the majority of patients, some studies report dissatisfaction after TKA to be as high as 20%. Among other things, pain catastrophising and self-efficacy are thought to compromise results of TKA. Implant manufacturers keep improving upon their designs in an attempt to improve functional outcomes. One of these novel knee systems is the Attune. To our knowledge, there are no clinical follow-up studies reporting results of the uncemented version. The main objective of this multicentre prospective observational study is to evaluate revision rate, complications, radiographic outcomes (i.e. alignment and radiolucent lines) and patient reported outcomes of the uncemented Attune mobile bearing TKA. Secondary objectives are (1) to assess physical function, return to sport and return to work after TKA and (2) to evaluate the long-term effect of preoperative psychological factors on satisfaction after TKA.

**Methods:**

All patients presenting in the participating centres with knee pathology warranting joint replacement therapy will be considered for inclusion, an absolute indication for cemented fixation is the only exclusion criterium. Evaluation of clinical and radiographic performance (e.g. radiolucent lines) is done at 6 weeks, 6 months, 1 year, 5 years and 10 years after surgery using validated patient reported outcome measures. Cumulative revision rates are calculated after 5 and 10 years using Kaplan–Meier methods. Physical function is assessed with performance based measurements before and 1 year after surgery. Return to sports is assessed using the Tegner and University of California Los Angeles (UCLA) activity rating scale before and 1 year after surgery. Return to work is evaluated by inviting patients of working age to complete a short questionnaire 1 year after surgery. Psychologic factors are assessed using questionnaires for pain catastrophising, pain self-efficacy and mental health before, 5 years and 10 years after surgery. Preoperative psychologic scores are correlated to functional outcomes.

**Discussion:**

The current study aims to report the clinical performance of a novel implant and can help provide insight in factors that play a role in satisfaction after TKA.

**Trial registration:**

ClinicalTrials.gov identifier: NCT04247672 (January 30, 2020)

## Background

Every year, 1.5 million total knee arthroplasties (TKA) are performed worldwide in patients whose joints have been severely affected by osteoarthritis, rheumatoid arthritis, or trauma, causing intense pain and loss of function. Due to the ageing society, these numbers are expected to increase with up to 43% by 2050 [1]. Even though joint replacement provides satisfactory and durable results for most patients, up to 20% are thought to still not be satisfied with their artificial joint [2]. Preoperative mental status and related pain coping have been identified as factors that may lead to dissatisfaction [3]. A review by Baert et al. identified catastrophic thinking and poor coping capabilities to predict more pain after TKA, whereas evidence on the impact on knee function remains conflicting in nature [3]. Most studies assess this influence only 1 year after surgery, with the study by Brander et al. prolonging the follow-up to 5 years [4]. Whether pain catastrophising remains a significant predictor of dissatisfaction at long-term follow-up remains to be elucidated.

Besides surgical performance, patient characteristics and expectation management [5], several knee systems have been developed in an attempt to improve functional outcomes. One of these models is the uncemented Attune knee system (DePuy, Warsaw, Indiana, USA), first implanted in October 2016. One of the landmark features is a gradually reducing radius in the geometry of the femoral component, more closely mimicking the anatomical patellofemoral joint and facilitating more natural femoral rollback during flexion [6–8]. Its predecessor (Low Contact Stress, DePuy, Warsaw, Indiana, USA) had excellent results [9], but it is still unclear whether the newer, more costly, Attune outperforms it. Comparisons of the cemented Attune with previous knee systems show promising results in terms of patellofemoral outcomes [10–13], but fail to demonstrate definitive superiority in terms of all patient reported outcomes [10, 11, 14–19]. An important note is that all cited studies compare posterior stabilized implants, which are significantly different with regards to patellofemoral kinematics compared to cruciate retaining designs [20]. Clinical superiority of the Attune tends to abate with longer follow-up, implicating a possible advantage in short-term recovery and return to activities. There is no follow-up study reporting the results of the uncemented Attune. Moreover, all previously cited studies report better patellofemoral outcomes with patellar resurfacing, making it still unclear whether the implicated superior design changes of the femoral component hold ground without patellar resurfacing. Due to the significant differences in costs, the proposed superiority needs more scientific scrutiny to justify the higher implant price.

The primary objective of the current multicentre prospective cohort study is to report survivorship, complications, radiographic outcomes (i.e. alignment and radiolucent lines) and patient reported outcome measures associated with the uncemented Attune mobile bearing (“rotating platform”) cruciate retaining knee system without patellar resurfacing. Secondary objectives are (1) assess return to activities (work and sports) after TKA and (2) analyse preoperative psychologic factors and their influence on patient satisfaction following TKA.

## Methods/Design

### Study design

The current study is a prospective cohort study. Patients will be recruited from high-volume TKA orthopaedic departments in the following 6 hospitals in the Netherlands:

- Spaarne Gasthuis, Hoofddorp (3 participating surgeons with extensive experience with uncemented fixation), coördinating site
- Bergman clinics, Rijswijk (2 participating surgeons with extensive experience with uncemented fixation)
- Medisch Spectrum Twente, Twente (2 participating surgeons with limited experience with uncemented fixation)
- Maastricht Universitair Medisch Centrum, Maastricht (2 participating surgeons with limited experience with uncemented fixation)
- Alrijne Ziekenhuis, Leiderdorp (2 participating surgeons with average experience with uncemented fixation [limited to unicondylar knee arthroplasty])
- Bravis Ziekenhuis, Bergen op Zoom (1 participating surgeon with extensive experience with uncemented fixation)

All surgeons with limited experience regarding uncemented TKA fixation are extensively trained by experienced colleagues and the implant manufacturer before participation in the study.

### Patient selection

The population will consist of patients with symptomatic knee pathology warranting joint replacement therapy (i.e. primary, secondary and traumatic osteoarthritic knees with or without previous arthroscopic or open knee surgery). To be eligible for inclusion, a subject must be between 21–90 years old and capable of understanding and complying with study procedures. A patient will be excluded if there is any absolute contra-indication for uncemented fixation at discretion of the surgeon (e.g. decreased bone stock/quality of the spongiosa), there is an indication for primary revision arthroplasty (e.g. stemmed components) or if the patient is unwilling to sign informed consent or comply with follow-up procedures. The patient is informed and recruited by the treating orthopaedic surgeon. After being informed verbally and in writing by the treating surgeon and the research nurse, the patient is given a mandatory reflection period of 14 days. Thereafter, the informed consent form is signed first by the patient and secondly by the surgeon or his/her designated staff (e.g. research nurse).

### Surgical procedure

All surgeons participating in the study are properly trained for the Attune knee system according to the instructions of the implant manufacturer (DePuy Synthes, Warsaw, Indiana, USA). To avoid bias, all participating surgeons will use the same surgical techniques. A tourniquet is never used and surgery is performed under spinal anaesthesia. A standard medial parapatellar incision with a medial arthrotomy is used. All knees are mechanically aligned with intramedullary (femur) and extramedullary (tibia) alignment guides. Gap balancing methods and ligament releases are used to balance the knee. An additional release of the posterolateral capsule is performed for fixed valgus knee deformities. Osteophytes are removed around the patella and it is radially circumcised with electrocauterization, a lateral release or facetectomy is performed when indicated by the surgeon. The patella is only resurfaced if strictly indicated by the surgeon thus it is not standard care. Local infiltration analgesia (LIA), active prevention of postoperative nausea and vomiting (PONV) and immediate postoperative mobilization under the supervision of a physical therapist are routinely offered to ensure fast rehabilitation. Thrombosis prophylaxis is according to the standard of care of the participating institution. Follow-up will be scheduled at 6 months, 1 year, 5 years and 10 years after surgery.

### Implant design

**Fig. 1:**
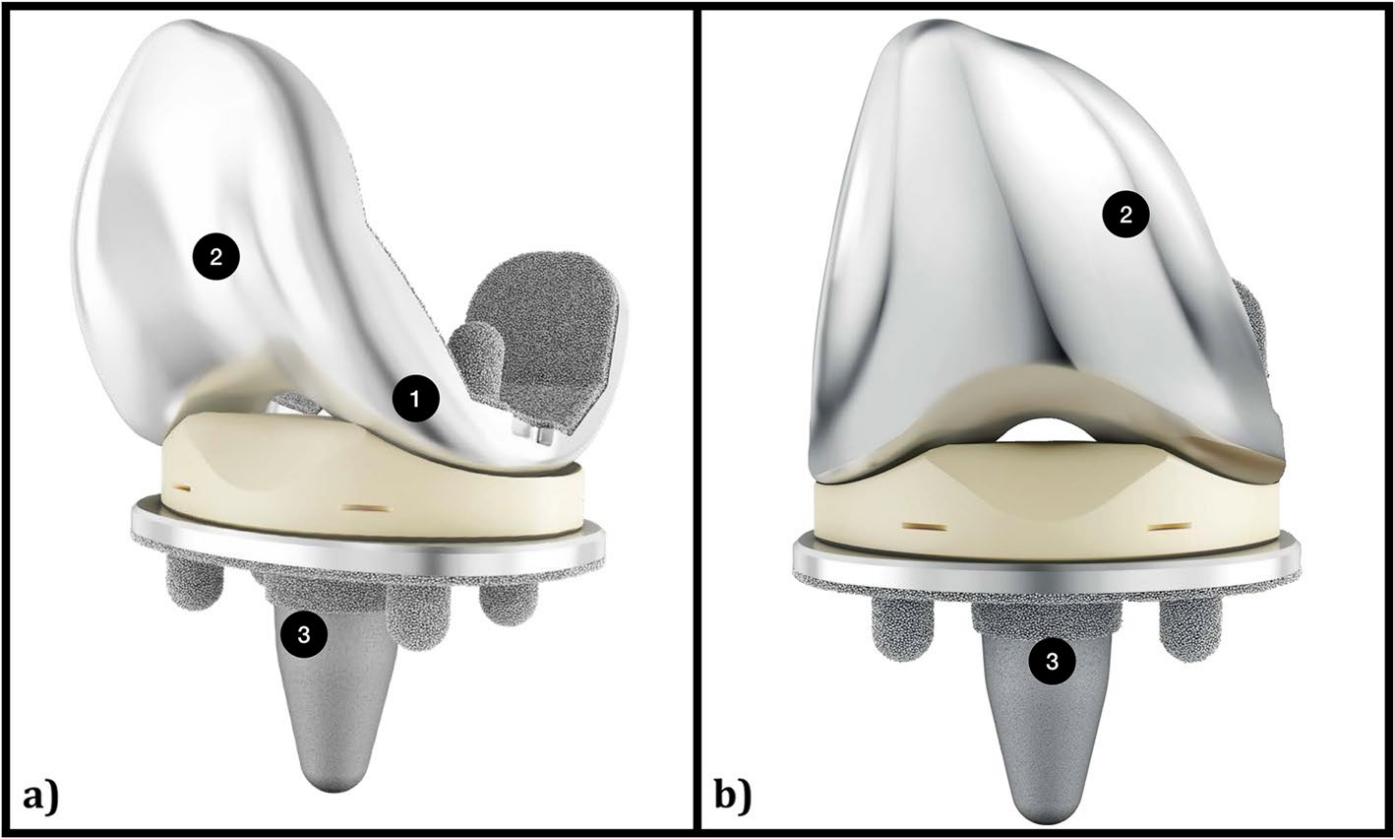
Oblique (**a**) and anteroposterior (**b**) view with landmark features of the uncemented Attune mobile bearing (“rotating platform”) implant (DePuy Synthes, Warsaw, Indiana, USA). A gradually reducing radius of the femoral component (1) allows for more stability during flexion. The patellofemoral surface of the femoral component mimic the original anatomy more closely (2), providing more natural kinematics and patellar tracking during knee flexion. A porous coating on the 4 pegs and proximal part of the central cone provides an ideal environment for fixation (3). Images provided courtesy of DePuy Synthes

All patients are receiving the uncemented, mobile bearing (“rotating platform”), cruciate retaining configuration of the Attune primary knee system (DePuy Synthes, Warsaw, Indiana, USA) (Fig. 1). The femoral component has been reshaped (trochlear groove accommodates patient variation in patellar tracking and a gradually reducing radius to provide anteroposterior stability during full range of motion). The tibial tray contains four pegs that are positioned radially around a central cone which, in synergy with an optimized porous coating pattern, reduces micromotion of the tibial tray and thus optimizes initial uncemented fixation. The size range has been expanded to accommodate patient variation and the polyethylene is better designed to deliver durable oxidative stability.

### Main outcomes – revision rate, complication rate, alignment, radiolucencies and PROMs

The main outcomes are revision rate, complication rate and patient reported outcome measures (PROMs) at 1 year, 5 years and 10 years after index surgery (Table 1). Revision is defined as any alteration to the implanted knee (e.g. implantation, explantation or replacement) including at least one component. Revision is further divided into major revisions (exchange or explantation of metal components) and minor revisions (exchange of insert or placement of patellar prosthesis).

**Table 1:** Study procedures and distribution of the different outcomes in the follow-up schedule

	Preoperative	6 months	1 year	5 years	10 years
Patient reported outcome measures	X	X	X	X	X
Return to sports and physical function					
Tegner / UCLA activity scale	X		X		
Performance based measurements	X		X		
Return to work			X		
Radiographic analysis					
Radiolucent lines		X	X	X	X
Alignment	X		X		
Psychologic factors	X		X	X	X

N.B. Patients are routinely seen at the 6 week follow-up mark in some of the participating centres, no study parameters are registered at this visit

Complications are represented by any adverse event related to the knee surgery arising in the postoperative period. Complications are either major (e.g. prosthetic joint infection, reoperation other than revision, bearing dislocation/spin-out, manipulation under anaesthesia, venous thrombo-embolism) or minor (e.g. superficial wound infection, delirium).

Alignment is assessed on long-leg full weight bearing bilateral conventional radiographs before surgery and at the 1 year follow-up mark according to the parameters proposed by Hadi et al. (Table 1) [21]. Alignment parameters that are assessed include: sagittal and coronal tibial angle (sTA and cTA); sagittal and coronal femoral angle (sFA and cFA); coronal tibio-femoral mechanical angle (cTFmA), coronal tibiofemoral anatomical angle (cTFaA). All radiographs are furthermore assessed for progressive radiolucent lines at all time points according to the Knee Society Total Knee Arthroplasty Roentgenographic Evaluation and Scoring System [22].

Patients are asked to complete questionnaires before surgery and at every follow-up visit, which consists of 6 validated PROMs (Table 1). The EuroQol 5D-5L is a validated questionnaire for general health [23]. The Oxford Knee Score (OKS) is a validated and extensively used 12-item questionnaire for the subjective evaluation of knee function and pain [24, 25]. The Forgotten Joint Score 12 (FJS-12) is a knee specific questionnaire to assess joint awareness during daily living and has less of a ceiling effect when compared to other knee specific PROMs (e.g. patients ‘forget’ about a well-functioning joint) [26, 27]. Patellofemoral pain and function will be assessed with the Kujala Anterior Knee Pain Scale (AKPS) [28–30]. Finally, patients are asked to rate their pain (during activity and rest) and satisfaction concerning the TKA on a Numeric Rating Scale (NRS). All questionnaires are supplemented with 2 anchor questions that measure change in pain and daily function since index surgery.

### Secondary outcome – return to sport and physical function

According to recommendations made in the systematic review by Witjes and colleagues, we will assess return to sport by validated questionnaires and assessment of physical performance [31]. Patients are asked what kind of sport they perform (and whether this has changed due to the surgery), frequency and when they restarted after TKA. The Tegner rating scale is a validated questionnaire to assess level of sports and will be used in the current study [32–34]. The University of California Los Angeles (UCLA) activity scale is furthermore validated and assessed for cross-cultural adaptation in the Dutch population according to the ISPOR manual (The Professional Society for Health Economics and Outcomes Research) [35, 36]. The UCLA and Tegner scale will be completed preoperative and 1 year postoperative (Table 1).

Secondly, objective physical function before and 1 year after surgery will be assessed using 3 performance based measurements (PBMs) recommended by the Osteoarthritis Research Society International (OARSI): 30 s chair test (30 s-CST), 40 m fast paced walk test (40 m-FPWT), and the stair climb test (SCT) [37]. The PBMs will be done according to a standardized protocol that will be followed in all participating centers.

### Secondary outcome – return to work

All patients of working age (18–67 in the Netherlands) are invited to complete questionnaires 1 year after the index surgery to determine (Table 1):

1. Whether or not patients return to their (original) work
2. How long after the index surgery they returned to work
3. Working hours per week
4. Physical job demands according to the US Department of Labor’s Dictionary of Occupational Titles (sedentary, light, medium, heavy, and very heavy) [38].

### Secondary outcome – psychological factors

Main psychological factors that are evaluated are pain coping, pain catastrophising and depression. Patients are invited to complete 3 validated questionnaires before surgery, at the 5 and 10 year follow-up mark (Table 1). The Pain Self Efficacy Questionnaire (PSEQ) asks patients to take their pain into account when rating their self-efficacy beliefs [39, 40]. The Patient Health Questionnaire (PHQ-2) is a two-item questionnaire that is an accurate screening tool for major depressive disorders [41, 42]. The Pain Catastrophizing Scale (PCS) explores catastrophic thinking and its influence on pain behaviour [43, 44].

### Statistical analysis

Sample size calculation was aimed at non-inferiority of the Attune knee compared to the LCS knee, the predecessor of the Attune. A non-inferiority margin of 1.5% was assumed. Survival data of the LCS was extracted from a meta-analysis on long-term survivorship studies of the LCS TKA (mobile bearing cemented and uncemented implants) [45]. Pooled 10 year survivorship of the LCS was 98.1% [45]. Based on α = 0.05, power (1-β) of 0.90 and both true proportion (p) and null hypothesis proportion (p0) of 98,1%, a sample size of *n* = 709 was calculated with a one-sided test [46, 47]. Taking drop- outs and death within 10 years into account (approximately 20–25%), we are aiming for inclusion of *n* = 900 knees.

All data will be collected in Research Manager (Cloud9 software, Deventer, Netherlands) and exported for analysis to SPSS Statistics 26.0 (IBM SPSS, New York, USA). Data is coded and the encryption key is stored on the local hospital server of the participating institution. Statistical analysis will be mainly descriptive. Baseline characteristics and results will be described as means with standard deviations (SD) or 95% confidence intervals (95% CI), medians with interquartile ranges (IQR) or frequencies with accompanying proportions. Revision rates will be calculated by use of Kaplan–Meier survival analysis and competing risk analysis, where death is considered a competing risk [48]. Multivariable Cox proportional hazard analyses will be performed to assess the influence of radiolucent lines and implant alignment on implant survival and hazard ratios (HR) with 95% CI will be calculated. Mixed model analysis for repeated measures will be used for assessment of change in PROMs scores during follow-up. Multivariate linear regression analysis will be used to assess the association of radiolucent lines, implant alignment and preoperative psychologic scores (PSEQ, PCS and PHQ-2) with PROMs after 1, 5 and 10 years after index surgery.

Descriptive statistics is provided on the proportion of patients that return to sport, what kind of sports they do (and whether this has changed due to surgery), the frequency and time to restart sports after TKA (in weeks). Improvements in Tegner score, UCLA activity scale and PBMs will be assessed with paired T-tests (or Wilcoxons Ranks sum test in case of non-parametric distribution). Descriptive statistics is provided on the proportion of patients of working age that return to work, time to restart work, working hours per week and physical job demands.

For all analyses, the significance level is set at 5% (*p* < 0.05).

### Data monitoring

The coordinating site (Spaarne Gasthuis) is responsible for data monitoring. Due to a very low additional risk the study brings, the medical ethics committee concluded that no data monitoring committee is necessary. All (serious) adverse events related to the implant are reported by the coordinating site to the medical ethical committee within 7 (serious adverse events) or 15 (adverse events) days.

## Discussion

Mode of implant fixation, cemented or uncemented, remains heavily debated among knee surgeons. Advantages of uncemented fixation include shorter surgery time and prevention of cement wear [49, 50]. Fear of aseptic loosening remains the main source of criticism for uncemented fixation [51]. High-level evidence fails to appoint superiority over both modes of fixation [50, 52–56], but registry and observational data slightly favours cemented implants in terms of durability [51, 52, 56]. One of the main drawbacks of registry studies is the fact that older uncemented implants (with known design flaws or absence of porous hydroxyapatite or tantalum coating) often skew reported survival of all uncemented implants [52, 56–60]. The current study can further contribute to this burden of evidence and determine long-term durability of the newest uncemented implant design in a large population.

Even though a highly successful procedure, a substantial group of patients remain dissatisfied after their TKA [61–65]. Besides distinct surgery specific factors affecting knee pain (e.g. severe malalignment, instability, infection, loosening), psychologic factors are known to play an important role in the perception of one’s artificial knee joint [66–70]. The current study can provide insight in the long-term impact of preoperative pain behaviour in a large population undergoing TKA, possibly predicting dissatisfaction. Interventions to modulate these pain believes before replacing the affected joint might provide useful in maximizing patient satisfaction after TKA [71].

There are several challenging aspects in the design of the current study. Minimizing loss to follow-up can prove difficult. Due to the highly successful nature of TKA, patients may not be willing to return for a clinical visit 10 years after surgery. Another deterrent to follow-up may be the high number of outcome measures recorded. Digital options for collection of the primary outcome measures (revision rate, complication rate and PROMs) can provide useful in this aspect and maximize data collection. The multicentre design always introduces bias due to the heterogeneity of surgical environment and hospital-specific factors. This is minimized by standardized surgical procedures and regulations already in place in all orthopaedic clinics (by the Dutch Orthopaedic Society). Nonetheless, a multicentre design also allows for generalisability of results and provides robust external validity for European health care environments. Furthermore, the role of industry funding should be considered as a possible source of bias for our study. However, the current study is investigator-initiated (i.e. protocol was constructed before the industry knew of its existence) and the role of the industry is as a funding body only. Therefore, there was no influence on methodology and there will be no influence on the results or their interpretation.

In conclusion, the current multicentre prospective observational cohort study aims at reporting the long-term clinical outcomes of a novel uncemented knee system. We furthermore attempt to provide information on return to work and sports, utilizing validated outcome scales supplemented with performance based measurements as a proxy for physical performance (as recommended by Witjes et al.) [31]. This information can be used to inform patients of their chances to return to sports and work after TKA. Finally, we also seek to shed light on the role of pain behaviour and mental status in the long-term patient satisfaction after TKA.

## Data Availability

The final dataset will only be accessible by qualified researchers from the coordinating site. The data is not available due to the lack thereof in this stage of the study.

## Abbreviations

TKA: Total knee arthroplasty
PBM: Performance based measurments
PROM: Patient reported outcome measure
EQ5D: EuroQol 5D
KOOS: Knee injury and osteoarthritis outcome scale
FJS-12: Forgotten joint score
NRS: Numeric rating scale
AKPS: Anterior knee pain scale
CFB: Change from baseline
PSEQ: Pain self-efficacy questionnaire
PCS: Pain catastrophizing scale
PHQ-2: Patient health questionnaire
